# Effects of high oleic full-fat soybean meal on broiler live performance, carcass and parts yield, and fatty acid composition of breast fillets

**DOI:** 10.1016/j.psj.2023.103399

**Published:** 2024-01-10

**Authors:** Muhammad Ali, Michael Joseph, Maria Camila Alfaro-Wisaquillo, Gustavo Adolfo Quintana-Ospina, Danny Patiño, Thien Vu, Lisa L. Dean, Ben Fallen, Rouf Mian, Earl Taliercio, Ondulla Toomer, Edgar Orlando Oviedo-Rondón

**Affiliations:** ⁎ Prestage Department of Poultry Science, North Carolina State University, Raleigh, NC 27695, USA; † Food Science & Market Quality and Handling Research Unit, Agricultural Research Service, United States Department of Agriculture, Raleigh, NC 27695, USA; ‡ Soybean and Nitrogen Fixation Research Unit, Agricultural Research Service, United States Department of Agriculture, Raleigh, NC 27695, USA; § Trouw Nutrition-Latin America, Ciudad de Guatemala, Guatemala

**Keywords:** broiler, meat fatty acid profile, high-oleic soybean, full-fat soybean meal

## Abstract

The effects of high oleic oil full-fat (**HO-FF**) soybean meal (**SBM**) on broiler meat quality could lead to value-added food products. This experiment evaluated the effects of dietary normal oleic extruded expelled (**NO-EE**), normal oleic full-fat (**NO-FF**), or HO-FF SBM on live performance, carcass and parts yield, and breast fatty acid composition. Diets were formulated to be isoenergetic and isonitrogenous. A total of 540 Ross-708 male broilers were raised on floor pens with 18 broilers/pen and 10 replicates/treatment. Data were analyzed in a completely randomized design. Chickens were fed with a starter (0–14 d), grower (15–35 d), or a finisher diet (36–47 d) up to 47 d. Chickens were weighed at 7, 14, 35, and 47 d. At 48 d, 4 broilers per pen were processed. Breast samples were collected and evaluated for quality and fatty acid content. Broilers fed diets with NO-EE were heavier (*P <* 0.05) than chickens fed diets with full-fat SBM (NO-FF and HO-FF) at d 7, 14, 35 while feed conversion ratio (**FCR**) of NO-EE was best (*P <* 0.05) at 7 and 47 d. Carcass yield was also higher for broilers fed NO-EE than the other treatments. Diet did not affect parts yield, breast meat color, cooking, drip loss, white stripping, or SM quality parameters. More breast fillets without wooden breast (score 1) were observed (*P <* 0.05) for NO-FF than the other 2 treatments. The breast meat fatty acid profile (g fatty acid/100 g of all fatty acids) was significantly affected (*P <* 0.001) by diet. Broilers fed the HO-FF SBM diet had 54 to 86% more oleic acid, 72.5% to 2.2 times less linoleic acid, and reduced stearic and palmitic acid levels in the breast meat than NO-FF and NO-EE. In conclusion, feeding HO-FF to broilers enriched the oleic acid content of their breast meat while reducing the saturated fatty acid content relative to the NO-FF and NO-EE treatment groups.

## INTRODUCTION

Seventy-seven percent of globally produced soy is utilized in animal nutrition, with poultry consuming 64 to 67% of USA-produced soybean meal (United Soybean Board, 2020). Traditionally, the oil is extracted from the seed, resulting in solvent-extracted soybean meal (**SE**-**SBM**), mainly used as a high-protein source with good digestibility for broiler diets. However, a full-fat soybean meal (**FFSB**) could replace these 2 feed ingredients in livestock production. Generally, FFSB is a good source of dietary protein and energy and can replace partially SE-SBM and vegetable oil in poultry rations (Mihandoost et al., 2021; Karimi et al., 2022).

Expansion and development of the soybean germplasm through breeding programs have led to the development of high-oleic (**HO**) soybean cultivars with a lipid profile of >75% oleic and approximately <1.5% linoleic acid as compared to NO soybeans that have a lipid profile of 18% oleic and 55% linoleic acid (Clemente and Cahoon, 2009). Studies have shown that elevation in oleic acid content, a monounsaturated fatty acid (**MUFA**), in oilseeds such as soybeans and peanuts has some advantages. It extends product shelf life by preventing oxidative rancidity of dietary fats within the feed or finished product as compared to prepared with normal-oleic (**NO**) oilseeds (Clemente and Cahoon, 2009; Martin et al., 2018; United Soybean Board, 2022)

A portion of the oil can be recovered from SB by applying mechanical pressure in expeller extraction (**EE**) processing. The utilization of FFSB has gained more popularity in SB-growing regions that lack oilseed-processing plants, as it avoids the cost of sending the SB to a large solvent-extraction plant and shipping the meal back. Some regions have significantly less supply of SE SBM because there are no large solvent extraction plants. These plants require a large capital investment and profits can be obtained only when the capacity is over 35,000 tons of soybean oil production (Cheng and Rosentrater, 2017). Hexane extraction has the highest environmental impact among soybean oil production methods due to the application of organic solvents (Cheng et al., 2018). An EE SBM may be an alternative protein and energy source to SE SBM or FFSB in those areas. The extrusion and expelling process produce some frictional heat but requires additional heat to create pressure for oil extraction and also destroy antinutritional factors, such as trypsin inhibitors (**TI**). Expelling extraction of soybean oil has the highest greenhouse gas and criteria pollutants emissions, among all oil extraction methodologies, because of the high-energy requirement for heat pressing processes (Cheng et al., 2018). The TI can affect broiler live performance through disrupting the intake, digestion, and absorption of proteins (Nitsan and Nir, 1977; Liener and Kakade, 1980). Heat can destroy most TI, so TI activity (**TIA**) may differ between SBM sources depending upon the processing method (Clarke and Wiseman, 2005).

An animal's dietary fatty acid profile is reflected in its meat tissue's fatty acid content, and chickens’ nutrition significantly affects their meat quality and safety (Mir et al., 2017). MUFA can improve the degree of unsaturation of intramuscular fat without any adverse effect on lipid oxidation linked with dietary polyunsaturated fatty acids **(PUFA**) (Rodríguez et al., 2005).

Poultry feeding trials with whole unblanched HO peanuts demonstrated that the meat and eggs from birds fed the HO peanuts had significantly higher levels of oleic MUFA content compared to normal-oleic fed broilers (Toomer et al*.*, 2019,2020a). Moreover, there is growing evidence that human diets enriched with MUFA can positively affect human cardiovascular health by lowering low-density lipoprotein cholesterol without lowering high-density lipoprotein cholesterol in blood plasma (Lichtenstein et al., 2006) while also reducing the susceptibility of low-density lipoprotein oxidation (Grundy, 1986; Roche, 2001). Consequently, the objective of this study was to evaluate the effect of dietary full-fat high oleic (**HO-FF**) SBM on live broiler performance, carcass and parts yield, and breast fatty acid composition in comparison to NO extruded expelled (**NO-EE**) defatted SBM and NO full-fat (**NO-FF**) SBM.

## MATERIALS AND METHODS

All procedures involving the broilers were approved by the North Carolina State University Institutional Animal Care and Use Committee (Approved Protocol # 21-145).

### Soybean preparation and analyses

Near isogenic lines of NO SB (<25% oleic acid, >7% linolenic, USDA NC-Roy) and a nongenetically modified HO SB (>75% oleic acid, <2.0% linolenic, 7.1% linoleic acid, USDA N16-1286 BC4 NIL) cultivars were bred by the USA Department of Agriculture, Agriculture Research Service, Soybean and Nitrogen Fixation Research Unit, ARS (SNFRU, Raleigh, NC, USA). These varieties were planted under similar agronomic conditions on the same farm. Upon harvesting, all foreign material was removed using an Eclipse 324 seed and grain cleaner (Seedburo, Equipment Company, Des Plaines, IL, USA), and all whole soybeans were dried to approximately 10% moisture using ambient temperature and natural air drying.

Soybean meal sub-samples were analyzed for mycotoxins using standard methodologies (vomitoxin, aflatoxin, fumonisin, ochratoxin, T-2 toxin, zearalenone), fatty acid composition using methodologies described by Toomer et al. (2019), and trypsin inhibitors at a commercial laboratory (ATC Scientific, Little Rock, AR, USA). Vomitoxin analysis was conducted using high-performance liquid chromatography (Schweighardt et al., 1980) with a DON test reference column purchased from Vicam (Watertown, MA, USA). AOAC official method 991.31 (2002) was utilized to determine aflatoxins B_1_, B_2_, G_1_, and G_2_ using immunoaffinity column cleanup with liquid chromatography. AOAC official method 2001.04 (2001) was utilized to determine fumonisin using high-performance liquid chromatography (**HPLC**) methods. Ochratoxin, T-2 toxin, and zearalenone levels were determined by modified HPLC- Mass Spectrometry (**MS**)/MS methods (Zhang et al., 2023) using respective HPLC reference columns purchased from Vicam (Watertown, MA, USA). Levels of mycotoxins in the soybean sub-samples were below the detection thresholds (Vomitoxin <0.10 ppm, Aflatoxin <2.0 ppb, Fumonisin <100.0 ppb, Ochratoxin <1.0 ppb, T-2 Toxin <25.0 ppb, Zearalenone <100.0 ppb) and the proximate composition and fatty acid analysis were within the expected parameters. The nutrient, amino acid, and energy values of the SB were obtained by using near-infrared spectroscopy (**NIRS**) calibration curves form the AMINONIR soybean package (Evonik Animal Nutrition, Hanau-Wolfgang, Germany) and wet chemistry methods described by Toomer et al. (2020b) at a commercial laboratory (ATC Scientific, Little Rock, AR, USA). These calibration curves provide information for all soybean products (Wiltafsky et al., 2019). These curves have been evaluated globally in the poultry industry and recently by Hack et al. (2023). A total of 15 replicates per soybean source were analyzed in NIRS to obtain average values for diet formulation.

### Production of soybean meals

Whole soybeans were used to produce NO-FF, HO-FF, and NO-EE SBM in a single screw dry extruder (InstaPro 2000 R, Grimes, Iowa, USA) at a die temperature of 155°C in a local commercial feed mill (Mule City Feeds, Benson, NC, USA). None of the soybeans were dehulled prior to extrusion. Diets were formulated on digestible amino acids (**AA**) content and to be isoenergetic and isonitrogenous. They contained identical digestible Lys, Thr, Val, total sulfur amino acids (**TSAA**), Ca, and available *P* in the 3 feeding phases. The nutrient compositions of the SBM are shown in Table 1. The ingredient composition (%) of each experimental diet is presented in Table 2, while energy and nutrient content are presented in Table 3. The isocaloric and isonitrogenous crumble starter diet fed from 0 to 14 d was formulated to have 3,000 kcal/kg and 22.83% **CP**. The pelleted grower diet fed from 15 to 35 d was formulated to have 3,100 kcal/kg and 20.70% CP. Finally, the pelleted finisher diet fed from 36–47 d was formulated to have 3,200 kcal/kg and 19.04% CP (Table 3). All diets met or exceeded Aviagen (2019) recommendations. Only starter and grower diets contained ionophores.

**Table 1:** Nutrient composition of soybean meals used in the broiler feeding trials.

	Soybean sources^1^		
Nutrient^2^	NO-EE	NO-FF	HO-FF
Protein, crude, %	43.80	38.31	38.18
Fat, crude, %	8.99	18.21	18.21
Fiber, crude, %	5.27	6.10	6.10
Calcium, %	0.20	0.28	0.28
Phosphorus total, %	0.57	0.48	0.48
Ash, %	6.41	5.52	5.52
Phosphorus available, % (calculated)	0.19	0.16	0.16
*Total amino acids*^3^			
Lysine, %	2.66	2.34	2.40
TSAA, %	1.21	1.06	1.13
Threonine, %	1.68	1.49	1.49
Valine, %	2.07	1.82	1.82
Leucine, %	3.33	2.86	2.80
Tryptophan, %	0.59	0.51	0.50
Trypsin inhibitor, mg/g^2^	7.46	11.02	11.02
*Digestible amino acids*^4^			
Lysine, %	2.36	2.03	2.09
Methionine, %	0.51	0.44	0.44
Cystine, %	0.49	0.40	0.45
TSAA, %	1.00	0.84	0.89
Threonine, %	1.41	1.24	1.24
Tryptophan, %	0.52	0.43	0.41
Isoleucine, %	1.82	1.51	1.49
Leucine, %	2.97	2.50	2.45
Valine, %	1.80	1.55	1.55
Histidine, %	0.99	0.87	0.88
Arginine, %	2.91	2.48	2.58
Phenylalanine, %	1.96	1.69	1.68
*Fatty acid profile*^2^			
Palmitic acid C16:0, %	0.80	1.93	1.20
Palmitoleic acid C16, %	0.01	0.03	0.02
Stearic acid C18:0, %	0.26	0.61	0.48
Oleic acid C18:1, %	1.40	3.12	11.13
Linoleic acid C18:2, %	3.79	9.55	1.71
Linolenic acid C18:3, %	0.59	1.45	0.02

1 NO-EE SB: normal oleic extruded expeller soybean; NO-FF: normal oleic and HO-FF, high oleic full-fat soybean meal; TSAA: total sulfur amino acids. All products were obtained from whole soybeans without dehulling.

2 The proximate analysis, trypsin inhibitor, mineral and fatty acid analyses were conducted by an AOAC-certified lab, ATC Scientific (Little Rock, AR, USA), *n* = 3.

3 Total amino acid content were determined by near-infrared spectroscopy (NIRS) using the AMINONIR RED package (Evonik Animal Nutrition, Hanau-Wolfgang, Germany;) for all soybean products (Wiltafsky et al., 2019), *n* = 15.

4 Digestible amino acids determined based on digestibility coefficients estimated by AMINODAT 5.0 (Evonik Animal Nutrition, Hanau-Wolfgang, Germany).

**Table 2:** Ingredient composition of normal oleic extruded expeller (**NO-EE**), normal oleic full-fat (**NO-FF**), or high oleic full-fat (**HO-FF**) soybean meal broiler experimental diets.

Ingredient (%)^1^	Starter (1–14 d)			Grower (15–35 d)			Finisher (36–47 d)		
	NO-EE	NO-FF	HO-FF	NO-EE	NO-FF	HO-FF	NO-EE	NO-FF	HO-FF
Corn	51.41	50.99	50.84	56.43	55.57	55.37	59.80	58.96	58.77
SE SBM	6.93	16.50	16.50	5.00	6.91	6.89	5.00	5.06	5.00
NO-EE SBM	32.23	0.00	0.00	28.92	0.00	0.00	24.60	0.00	0.00
NO-FF SBM	0.00	25.14	0.00	0.00	30.85	0.00	0.00	28.20	0.00
HO-FF SBM	0.00	0.00	25.38	0.00	0.00	31.12	0.00	0.00	28.50
DDGS	3.00	3.00	2.95	3.00	3.00	3.00	3.00	3.00	3.00
Poultry fat	1.96	0.00	0.00	2.90	0.00	0.00	4.16	1.42	1.42
Limestone fine	1.44	1.35	1.35	1.17	1.08	1.08	1.07	0.99	0.99
Dicalcium phosphate	1.13	1.11	1.11	0.94	0.94	0.94	0.81	0.81	0.81
DL-methionine	0.38	0.38	0.37	0.30	0.32	0.30	0.28	0.29	0.27
Sodium bicarbonate	0.26	0.27	0.27	0.20	0.21	0.20	0.25	0.26	0.25
L-lysine	0.27	0.28	0.25	0.19	0.20	0.17	0.18	0.19	0.17
Salt, plain (NaCl)	0.29	0.27	0.28	0.31	0.29	0.30	0.27	0.26	0.26
Mineral premix^2^	0.20	0.20	0.20	0.20	0.20	0.20	0.20	0.20	0.20
Choline chloride 60	0.18	0.18	0.18	0.18	0.18	0.18	0.18	0.18	0.18
L-threonine	0.16	0.15	0.15	0.09	0.09	0.08	0.07	0.07	0.07
Vitamin premix^3^	0.10	0.10	0.10	0.10	0.10	0.10	0.10	0.10	0.10
Coccidiostat^4^	0.05	0.05	0.05	0.05	0.05	0.05	0.00	0.00	0.00
Phytase^5^	0.02	0.02	0.02	0.02	0.02	0.02	0.02	0.02	0.02

1 SE SBM: solvent extracted soybean meal; SBM: soybean meal; NO-EE: normal oleic extruded expeller; NO-FF: normal oleic full-fat; HO-FF: high oleic full-fat; DDGS: dried distillers’ grains with solubles; NO: normal oleic; HO: high oleic.

2 Trace minerals provided per kg of premix: manganese (Mn SO4), 60 g; zinc (ZnSO4), 60 g; iron (FeSO4), 40 g; copper (CuSO4), 5 g; iodine (Ca(IO3)2),1.25 g.

3 Vitamins provided per kg of premix: vitamin A, 13,227,513 IU; vitamin D3, 3,968,253 IU; vitamin E, 66,137 IU; vitamin B12, 39.6 mg; riboflavin, 13,227 mg; niacin, 110,229 mg; d-pantothenic acid, 22,045 mg; menadione, 3,968 mg; folic acid, 2,204 mg; vitamin B6, 7,936 mg; thiamine, 3,968 mg; biotin, 253.5 mg.

4 Coban 90 (Monensin), Elanco Animal Health, Greenfield, IN, at 500 g/ton.

5 Natuphos E (500 FTU/kg, 50 g/ton FTU).

**Table 3:** Nutrient analysis of normal oleic extruded expeller (**NO-EE**), normal oleic full-fat (**NO-FF**), or high oleic full-fat (**HO-FF**) soybean meal broiler experimental diets (formulated values).^1^

Nutrient name^1^	Starter (1–14 d)			Grower (15–35 d)			Finisher (36–47 d)		
	NO-EE	NO-FF	HOFF	NO-EE	NO-FF	HO-FF	NO-EE	NO-FF	HO-FF
M.E. poultry, kcal/kg	3,000	3,000	3,000	3,100	3,100	3,100	3,200	3,200	3,200
Protein, crude, %	22.83	22.83	22.83	20.70	20.70	20.70	19.04	19.04	19.04
Protein, crude, %^1^ analyzed	21.50	22.29	22.70	20.85	20.53	20.60	19.14	19.00	18.98
Fat, crude, %	7.39	7.36	7.39	8.18	8.34	8.38	9.18	9.35	9.39
Fat, crude, %^1^ analyzed	6.16	7.09	6.98	7.53	8.10	8.33	7.97	8.53	8.93
Fiber, crude, %	2.93	3.12	3.13	2.76	3.18	3.19	2.59	3.00	3.01
Calcium, %	1.00	1.00	1.00	0.85	0.85	0.85	0.78	0.78	0.78
Phosphorus total, %	0.57	0.56	0.57	0.51	0.51	0.51	0.47	0.47	0.47
Ash, %	5.65	5.52	5.53	4.95	4.83	4.84	4.50	4.40	4.41
*P* available, % (calculated)	0.46	0.46	0.46	0.42	0.42	0.42	0.39	0.39	0.39
*Total amino acids*^2^									
Lysine, %	1.41	1.42	1.42	1.22	1.23	1.23	1.11	1.12	1.12
Total sulfur amino acids, %	1.05	1.06	1.06	0.93	0.94	0.95	0.87	0.88	0.88
Threonine, %	0.99	1.00	1.00	0.86	0.86	0.86	0.87	0.88	0.88
Valine, %	1.05	1.05	1.06	0.96	0.96	0.97	0.88	0.89	0.89
Leucine, %	1.91	1.89	1.88	1.78	1.76	1.74	1.67	1.65	1.64
Tryptophan, %	0.27	0.27	0.27	0.24	0.24	0.24	0.22	0.22	0.22
Trypsin inhibitor activity, mg/g^1^	2.47	2.93	2.95	2.21	3.47	3.50	1.88	3.16	3.19
*Digestible amino acids*^3^									
Lysine, %	1.28	1.28	1.28	1.09	1.09	1.09	0.99	0.99	0.99
Methionine, %	0.67	0.67	0.66	0.58	0.59	0.57	0.53	0.54	0.53
TSAA, %	0.95	0.95	0.95	0.84	0.84	0.84	0.78	0.78	0.78
Threonine, %	0.86	0.86	0.86	0.73	0.73	0.73	0.66	0.66	0.66
Tryptophan, %	0.24	0.23	0.23	0.21	0.20	0.20	0.19	0.18	0.18
Isoleucine, %	0.88	0.86	0.85	0.79	0.77	0.76	0.72	0.70	0.70
Leucine, %	1.83	1.81	1.79	1.62	1.58	1.57	1.52	1.49	1.48
Valine, %	0.95	0.95	0.95	0.84	0.83	0.83	0.77	0.77	0.77
Arginine, %	1.37	1.36	1.39	1.23	1.21	1.25	1.11	1.10	1.13
*Fatty acids*^1^									
Palmitic acid C16:0, %	0.30	0.60	0.42	0.26	0.64	0.42	0.23	0.58	0.38
Palmitoleic acid C16, %	0.00	0.01	0.01	0.01	0.01	0.01	0.00	0.01	0.01
Stearic acid C18:0. %	0.10	0.18	0.15	0.08	0.20	0.16	0.07	0.18	0.15
Oleic acid C18:1, %	0.50	0.90	2.94	0.44	1.01	3.51	0.38	0.91	3.20
Linoleic acid C18:2, %	1.41	2.48	0.879	1.23	3.13	0.71	1.06	2.83	0.62
Linolenic acid C18:3, %	0.22	0.43	0.19	0.19	0.48	0.18	0.17	0.43	0.16

ME = metabolizable energy.

1 Three finished feed samples of each experimental broiler diet were analyzed by wet chemistry. The proximate analysis (Analyzed), trypsin inhibitor, mineral, and fatty acid analyses were conducted by an AOAC-certified lab, ATC Scientific (Little Rock, AR, USA). Since lab results had good agreement (92%) with formulated values, these are presented.

2 The total amino acid content of soybean meal sources, corn and DDGS were determined by near-infrared spectroscopy (NIRS) using the AMINONIR RED package (Evonik Animal Nutrition, Hanau-Wolfgang, Germany; Wiltafsky et al., 2019), *n* = 15.

3 The digestible amino acids of all products were determined based on digestibility coefficients estimated by AMINODAT 5.0 (Evonik Animal Nutrition, Hanau-Wolfgang, Germany).

All diets contained the same corn, dried distillers’ grains with solubles (**DDGS**), oil source, and phytase at similar levels. Nutrient analysis of these ingredients was performed before mixing each dietary phase (starter, grower, and finisher). All diets were crumbled (starter) or pelleted at 85 °C while corn particle size was kept between 700 and 800 µm for the starter and 800 to 900 µm for the grower and finisher. All experimental diets were analyzed in triplicate for proximate and fatty acid analysis using methods described by Toomer et al. (2019,2020b) and determined to be free of aflatoxins using the aforementioned methods by Schweighardt et al., 1980; AOAC method 2001.04, 2001; AOAC method 991.31, 2002; Zhang et al., 2023 at a commercial laboratory (ATC Scientific, Little Rock, AR, USA). Results indicated good agreement (92%) with formulated values and these are presented in Table 3.

### Experimental design and animal husbandry

A total of 540 chicks of Ross-708 were hatched with 85% hatchability, feather-sexed, and males were placed in 30-floor pens (122 × 188 × 82 cm^3^) at the Chicken Education Unit at North Carolina State University (Raleigh, NC, USA) with 10 replicates per treatment. Pine shavings were used as litter. All pens were supplied with one tubular feeder and one belt drinker, while supplemental feeders and drinkers were used for the first 7 d of the experiment. All birds were raised on floor pens until 47 d of age and provided *ad libitum* one of the 3 dietary treatments: NO-EE (control), NO-FF, and HO-FF. Water was provided *ad libitum* to all broilers during the experiment. All chicks were raised in a poultry house with negative pressure tunnel ventilation, evaporative cooling pads, and a temperature-controlled environment. The initial temperature during brooding was set at around 32.2°C, while from d 2 to 7, it was kept between 29 and 32°C. The temperature was reduced by 2°C every week as the broilers aged. From d 22 to the end of the experiment, the temperature set point was between 20 and 23°C.

### Data and sample collection

Pen BW and feed intake (**FI**) were recorded at 7, 14, 35, and 47 d of age, while BW gain, feed conversion ratio (**FCR**), and adjusted FCR (adjusted by mortality weight) were calculated. All chickens were individually weighed at 47 d to obtain flock uniformity data. At 48 d, 2 broilers above and 2 broilers below the average pen weight were selected within each replicate for a total of 40 chickens per treatment. Feed was withdrawn 12 h before termination, and selected broilers were processed at the NC State University processing plant (Raleigh, NC, USA). Broilers were weighed and slaughtered by exsanguination and bled for 90 s. Carcass dressing was completed by removing the liver, gizzard, heart, oil gland, crop, proventriculus, lungs, and viscera. Upon evisceration, carcasses were air-chilled in a walk-in cooler for 6 h to start a manual deboning on stationary cones. Leg quarters, breast fillets (*Pectoralis major*), breast tenders (*Pectoralis minor*), wings, and racks (thoracic vertebrae, ribs, clavicle, and sternum) with skin were removed and weighed. Subsequently, the *Pectoralis major* and *Pectoralis minor* muscles were stored at −20°C for further analysis.

Meat quality was assessed by evaluating drip loss, cook loss, color, pH, and pectoral myopathies, including wooden breast (**WB**), white striping (**WS**), and spaghetti muscle (**SM**). Subject matter experts scored poultry breast myopathies to prevent subjective variations due to the evaluator. White striping was scored on a scale of 0 to 3, with a score of 0 = indicating no white striping, score 1 = white striping, score 2 = moderate white striping, and score 3 = severe white striping (Bailey et al., 2015). Wooden breast (level of hardness) was scored on a 4-point scale with score of 0 = absence of WB or hardness, score 1 = mild hardening in the upper sections of breast fillet, score 2 = moderate hardening in the upper and/or lower part of the fillet, score 3 = severe hardening in the breast fillet, score 4 = severe hardening with hemorrhagic lesions, increased volume and presence of yellow fluid in breast fillet (Vieira et al., 2021). The SM muscle was scored as a yes (presence) or no (absence) using Che et al. (2022) as a reference.

Drip loss was evaluated by weighing breast fillets and then hanging them from a plastic hook in a refrigerator at 4 to 6°C for 24 h. After the given time, each fillet was weighed again carefully, and the difference was determined. Cook loss was evaluated by measuring the differences in the weight of breast fillets before and after cooking to compare fluid losses in the meat between dietary treatments. Breast fillets were placed in aluminum pans and cooked in a forced-air oven (SilverStar Southbend, Model SLES/10sc, gas type, NC, USA). The target internal temperature of 75°C was achieved in approximately 35 min and assessed with a Therma Plus thermocouple with a 10 cm needle temperature probe (ThermoWorks Model 221-071, American Fork, UT, USA). Subsequently, the breast fillets were allowed to cool at room temperature and re-weighed to assess the difference before and after cooking.

The breast meat fillet pH was determined using a portable pH meter (Oakton-Eutech Instruments waterproof pH Tester 30, Cole-Parmer, Vernon Hills, IL, USA) at 6 and 24 h after processing. Meat color values (L* lightness, a* redness, and b* yellowness) of the breast were assessed by a colorimeter, Minolta Chroma Meter CR-400 (Konica Minolta Sensing, Inc., Japan). Two poultry meat experts conducted the sensory evaluation and scoring to identify the presence of WB (1–4), WS (0–3), or SM (presence or absence).

Fatty acid analysis was conducted on the breast fillets (*Pectoralis major*) and breast tenders (*Pectoralis minor*) samples stored at -20°C for approximately 2 wk. The chicken breast samples were thawed overnight at 4°C and homogenized using a commercial food processor (Blixer Model 6, Robot Coupe, Jackson, MS, USA) using modified methods described in Toomer et al. (2019). All samples were extracted using a modified Folch procedure in which samples of 10 g were weighed into 250 mL centrifuge bottles, then the process outlined in Folch et al. (1957) was followed. The fat content of the original meat sample was quantified according to the equation:

Fatinsamples(%)=Residueweight(g)÷Sampleweight(g)*100

The fatty acid composition of meat samples was determined following methods outlined by Bannon et al. (1982). Gas-liquid chromatography was conducted using a PerkinElmer AutoSystem XL gas chromatograph with an autosampler (PerkinElmer Inc., Norwalk, CT, USA) and a BPX70 capillary column (SGE Technologies, Merseyside, UK) of 30 m length, 0.25 mm inside diameter, and 0.25 µm film thickness. A commercial standard of fatty acid methyl esters (FIM-FAME-6, Matreya LLC, State College, PA, USA) was used to compare the retention times of compounds for identification. Quantitative analysis following the identification and normalization of peaks was accomplished following Official Method Ce 1-62 of the American Oil Chemists’ Society (AOCS, 2005).

### Statistical analysis

Pen served as the experimental unit for live performance, carcass and meat quality parameters. Four chickens were processed for carcass, cut-up parts, and meat quality evaluations. Their data was averaged for statistical analyses. Prior to statistical analyses, the distribution platform of JMP was used to verify normality. Any outliers, determined as 3 times the root mean square error (**RMSE**) plus or minus the mean of the response, were removed from the statistical analysis. Data were analyzed in a completely randomized design with 3 treatments and 10 replicates per treatment using 2-way ANOVA, while mean separation was accomplished using Tukey's tests at the significance level of *P <* 0.05. The arcsine transformation was used for all percentage data before analysis. Data were analyzed using the JMP Pro 15 software (SAS Institute, Inc., Cary, NC, USA).

## RESULTS

### Live performance

Live performance results of broilers from 0 to 7 d and 7 to 14 d are shown in Table 4, while from 0 to 35 d and 47 d are shown in Table 5. Broilers fed the NO-FF and HO-FF experimental diets had significantly lower BW and BWG relative to broilers fed the NO-EE experimental diet from 0 to 7 (*P* < 0.01) 0 to 14 d (*P* < 0.001) and 0 to 35 d (*P* < 0.05). The FCR was better (*P* < 0.05) in broilers fed NO-EE than in those fed HO-FF SB during the first 7 d (Table 4) and the adjusted FCR by mortality weight in the whole experiment (0–47 d), as shown in Table 5, while chickens fed diets containing NO-FF SB were intermediate. However, no effect (*P* > 0.05) on FCR was detected at 14 d, and from 0 to 35 d the adjusted FCR was better (*P <* 0.001) for broilers fed the NO-EE than those fed the NO-FF and HO-FF SB diets (Table 5).

**Table 4:** Effect of soybean meal sources on the live performance of Ross 708 male broilers from 0 to 7 d and 0 to 14 d of age on floor pens.

Treatment^1^	BW	BWG	FI	FCR
	—————g—————		———g:g———	
*0–7 d*				
NO-EE SB	176^a^	132^a^	152	1.152^b^
NO-FF SB	167^b^	123^b^	147	1.199^a,b^
HO-FF SB	166^b^	122^b^	149	1.225^a^
SEM	2	2	2	0.019
CV %	4.10	5.51	3.49	5.02
*P values*	0.005	0.005	0.122	0.033
*0–14 d*				
NO-EE SB	526^a^	483^a^	558^a^	1.146
NO-FF SB	497^b^	454^b^	529^b^	1.155
HO-FF SB	508^b^	465^b^	541^a,b^	1.165
SEM	4	4	6	0.007
CV %	2.37	2.58	3.51	1.81
*P values*	<0.001	<0.001	0.008	0.150

1 NO-EE SB, normal oleic extruded expeller soybean; NO-FF SB, normal oleic full-fat soybean; HO-FF SB, high oleic full-fat soybean; BW, body weight; BWG, body weight gain; FI, feed intake Means correspond to average of 10-floor pens per treatment.

a,b Means that do not share superscript letters in a column are significantly different (*P <* 0.05) by Tukey's test.

**Table 5:** Effect of soybean meal sources on the live performance of Ross 708 male broilers from 0 to 35 d and 0 to 47 d of age on floor pens.

Treatment^1^	BW	BWG	FI	Adj FCR^2^	Mortality	Flock BW uniformity
	—————————g—————————			——g:g——	———%———	———CV %———
*0*–*35 d*						
NO-EE SB	2,494^a^	2,449^a^	3,306^a^	1.350^b^	2.23	
NO-FF SB	2,404^b^	2,360^b^	3,178^b^	1.347^b^	1.11	
HO-FF SB	2,400^b^	2,356^b^	3,272^a,^^b^	1.389^a^	0.56	
SEM	28	28	34	0.006	1.05	
CV %	3.59	3.65	3.33	1.5		
*P* values	0.037	0.04	0.036	<0.001	0.398	
*0*–*47 d*						
NO-EE SB	3,603	3,558	5,568	1.532^b^	3.89	13.11
NO-FF SB	3,522	3,477	5,416	1.537a,b	3.33	12.42
HO-FF SB	3,545	3,499	5,677	1.603^a^	6.11	11.11
SEM	36.03	36.31	89.37	0.019	1.77	0.76
CV %	3.15	3.21	5.09	3.77		10.69
*P* values	0.291	0.295	0.136	0.026	0.467	0.177

1 NO-EE SB, normal oleic extruded expeller soybean; NO-FF SB, normal oleic full-fat soybean; HO-FF SB, high oleic full-fat soybean; BW, body weight; BWG, body weight gain; FI, feed intake. Means correspond to the average of 10-floor pens per treatment.

2 FCR was adjusted by mortality weights.

a,b Means that do not share superscript letters in a column are significantly different (*P <* 0.05) by Tukey's test.

No treatment effects (*P >* 0.05) were observed on FI at 0 to 7 d and 0 to 47 d. Broilers fed the NO-EE experimental diet had higher FI than broilers fed the NO-FF experimental diets between 0 and 14 d (*P* < 0.01), and 0 to 35 d (*P* < 0.05), with an intermediate FI for broilers fed the HO-FF diets. There were no treatment effects on flock uniformity and total mortality (*P* > 0.05) at 47 d (Table 5). However, the total mortality of chickens fed the diets containing HO-FF SB was 57% and 83% higher than in groups of chickens fed diets including NO-EE and NO-FF SB. No mortality was observed during the first 14 d of age. The higher mortality in the HO-FF SB occurred mainly during the last wk of the experiment in 4 pens due to heat stress and consequently adjusted FCR was used.

### Carcass and component parts yield

Broiler carcass and parts yield (%) are shown in Table 6. Carcass yield was higher (*P <* 0.05) in broilers fed the NO-EE experimental diet in comparison to the HO-FF experimental diet (77.42% vs*.* 76.54%), while the NO-FF diet was intermediate (77.02%). There were no effects (*P >* 0.05) of dietary treatments on the carcass part yield, including legs, wings, *Pectoralis major, Pectoralis minor*, breast and rack, and skin. Also, no effects (*P >* 0.05) of SBM source were detected on abdominal fat and or visceral organs (liver, pancreas, intestine, gizzard + proventriculus, spleen) on broilers at 47 d (Table 7).

**Table 6:** Effect of soybean meal sources on carcass and components parts yield (%) of Ross 708 male broilers at 47 d of age.

		Cut-up parts					
Treatment^1^	Carcass	Legs	Wings	*Pectoralis major*	*Pectoralis minor*	Breast	Rack + skin
	————————————————————%————————————————————						
NO-EE SB	77.42^a^	30.13	9.35	32.04	6.51	38.88	21.58
NO-FF SB	77.02 a,b	30.84	9.42	31.43	6.68	38.08	21.61
HO-FF SB	76.54^b^	30.63	9.40	31.30	6.65	37.94	22.02
SEM	0.22	0.22	0.10	0.30	0.07	0.31	0.25
CV %	1.52	2.81	2.73	3.52	4.67	3.03	3.27
Source of variation	———————————————————*P* values———————————————————						
Treatment	0.027	0.076	0.885	0.193	0.249	0.099	0.383

1 NO-EE SB, normal oleic extruded expeller soybean; NO-FF SB, normal oleic full-fat soybean; HO-FF SB, high oleic full-fat soybean. Means correspond to 40 chickens per treatment, 4 per replicate pen within one standard deviation of the average pen weight at 47 d.

a–b Means in a column not sharing a common superscript are significantly different (*P < 0.05*) by Student *t* or Tukey's test.

**Table 7:** Effect of soybean meal sources on fat and visceral organs (%) of Ross 708 male broilers at 47 d of age.

Treatment^1^	Abdominal fat	Liver	Pancreas	Intestine	Gizzard + proventriculus	Spleen
	————————————————————————%————————————————————————					
NO-EE SB	1.35	1.55	0.16	2.88	1.63	0.12
NO-FF SB	1.36	1.59	0.18	3.04	1.61	0.12
HO-FF SB	1.36	1.62	0.18	2.86	1.65	0.12
SEM	0.10	0.05	0.01	0.09	0.06	0.01
CV %	24.29	5.85	7.72	6.86	8.51	12.35
Source of variation	——————————————————————*P values*——————————————————————					
Treatment	0.910	0.663	0.093	0.337	0.887	0.942

1 NO-EE SB, normal oleic extruded expeller soybean; NO-FF SB, normal oleic full-fat soybean; HO-FF SB, high oleic full-fat soybean. Means correspond to 40 chickens per treatment, 4 per replicate pen within one standard deviation of the average pen weight at 47 d.

### Meat quality and meat fatty acid profile

No dietary treatment effects (*P >* 0.05) were observed on most of the meat quality parameters measured, which included breast pH at 6 h, color parameters (*L, a, b*), drip loss, and cooking loss (Table 8). However, the breast meat of broilers fed the NO-FF SB had a higher (*P* < 0.05) ultimate pH 24 h postmortem than the HO-FF SB. A higher incidence (*P <* 0.05) of normal fillets (WB score 1) for broilers fed the NO-FF diets than the other treatment groups, and a tendency (*P* = 0.065) for lower average WB was observed in chickens fed NO-FF SB. However, the SBM source did not affect (*P* > 0.05) WS or SM (Table 9).

**Table 8:** Effects of soybean meal sources on breast meat pH and color parameters of Ross 708 male broilers at 47 d of age.

	pH		Color parameters				
Dietary treatment^1^	6 h	24 h	*L*	*a*	*b*	Dripping loss	Cooking loss
						—————%—————	
NO-EE SB	5.81	5.90a,b	48.26	2.36	5.52	1.38	17.95
NO-FF SB	5.84	5.94^a^	47.66	2.33	5.00	1.40	18.03
HO-FF SB	5.80	5.89^b^	48.15	2.10	5.44	1.41	18.87
SEM	0.02	0.01	0.41	0.17	0.20	0.10	0.46
CV %	1.50	1.48	4.61	37.16	21.94	16.13	8.61
Source of variation	———————————————————————*P* values———————————————————————						
Treatment	0.268	0.045	0.559	0.504	0.168	0.912	0.456

1 NO-EE SB, normal oleic extruded expeller soybean; NO-FF SB, normal oleic full-fat soybean; HO-FF SB, high oleic full-fat soybean. Means correspond to 40 breast samples per treatment, 4 broilers per replicate pen within one standard deviation of the average pen weight at 47 d.

a,b Means that do not share superscript letters in a column are significantly different (*P <* 0.05) by Tukey's test.

**Table 9:** Effect of soybean meal sources on wooden breast (**WB**), white-stripping (**WS**), and the incidence of spaghetti muscle (**SM**) myopathies in Ross 708 male broilers at 47 d of age.

		WB score^2^				WS score				SM incidence (%)^3^	
Treatment^1^	WB average score	1	2	3	4	0	1	2	3	Yes	No
NO-EE SB	2.85^a^	2.50^b^	35.00	37.50	25.00	2.50	32.50	37.50	27.50	15.00	85.00
NO-FF SB	2.48^b^	17.50^a^	30.00	40.00	12.50	10.00	42.50	22.50	25.00	10.00	90.00
HO-FF SB	2.60a,b	5.00^b^	42.50	40.00	12.50	12.50	40.00	37.50	10.00	22.50	77.50
SEM	0.14	0.04	0.08	0.08	0.06	0.04	0.08	0.07	0.06		
Source of variation		————————————————————*P* value————————————————————									
Treatment	0.075	0.039	0.503	0.966	0.239	0.188	0.630	0.242	0.092	0.305	

1 NO-EE SB, normal oleic extruded expeller soybean; NO-FF SB, normal oleic full-fat soybean; HO-FF SB, high oleic full-fat soybean. White striping (WS) scale of 0 to 3, score 0 = no WS, score 1 = mild WS, score 2 = moderate WS, score 3 = severe WS. Means correspond to 40 breast samples per treatment, 4 per replicate pen with broilers within one standard deviation of the average pen weight at 47 d.

2 Wooden breast (WB) 4-point scale, score 0 = absence of WB, score 1 = mild hardening in the upper sections of breast fillet, score 2 = moderate hardening in the upper and/or lower part of the fillet, score 3 = severe hardening in the breast fillet, score 4 = severe hardening with hemorrhagic lesions, increased volume, and presence of yellow fluid in breast fillet.

3 Spaghetti muscle (SM) was scored as a yes (presence) or no (absence).

a–b Means in a column not sharing a common superscript are significantly different (*P < 0.05*) by Student *t* or Tukey's test.

Table 10 presents the effect of SBM source on the breast meat fatty acid profile (g fatty acid per 100 g of all fatty acids). Broilers fed diets containing HO-FF had 54 to 86% more oleic acid (*P <* 0.001) in their *Pectoralis major* and *Pectoralis minor* breast meat than broilers fed the other treatments. Other MUFA acids like palmitoleic (16:1 *trans*) and gondoic acid levels were also higher (*P <* 0.01) in the breast meat of broilers fed the HO-FF diets compared to the other 2 treatments. While palmitoleic (16:1 *cis*) and myristoleic acid were reduced (*P* < 0.001) in broilers fed FFSB, and lower in HO than in NO-FF SB.

**Table 10:** Effect of soybean sources on the profile of fatty acids in *Pectoralis* muscles of broilers raised until 47 d of age.^1^

	*Pectoralis major*						*Pectoralis minor*					
Fatty acids	NO-EE	NO-FF	HO-FF	SEM	CV%	*P* value	NO-EE	NO-FF	HO-FF	SEM	CV%	*P* value
*Monounsaturated* (MUFA)	———————%———————						———————%———————					
Oleic acid (18:1, *cis*)	31.50^b^	26.57^c^	48.85^a^	0.50	6.2	<0.001	33.27^b^	27.50^c^	51.33^a^	0.36	3.3	<0.001
Palmitoleic acid (16:1, *cis*)	3.48^a^	2.12^c^	2.56^b^	0.09	16.3	<0.001	3.92^a^	2.37^c^	2.80^b^	0.10	16.4	<0.001
Palmitoleic acid (16:1, *trans*)	0.34^a^	0.23^b^	0.41^a^	0.03	42.8	0.001	0.34^b^	0.26^b^	0.47^a^	0.03	36.9	<0.001
Pentadecanoic acid (15:1, *cis*)	1.21	1.23	1.09	0.08	42.9	0.484	0.69	0.77	0.82	0.06	42.2	0.301
Myristoleic acid (14:1, *cis*)	0.09^a^	0.04^b^	0.06^b^	0.01	51.1	<0.001	0.10^a^	0.06^c^	0.08^b^	0.00	24.9	<0.001
Heptadecenoic acid (17:1, *cis*)	0.35	0.345	0.37	0.02	46.6	0.782	0.26	0.26	0.30	0.02	40.0	0.120
Gondoic acid (20:1)	0.28^b^	0.24^c^	0.42^a^	0.01	19.4	<0.001	0.28^b^	0.24^c^	0.41^a^	0.01	20.5	<0.001
*Polyunsaturated* (PUFA)												
Linoleic acid (18:2, *cis*)	25.66^b^	32.61^a^	14.87^c^	0.33	5.7	<0.001	26.72^b^	34.64^a^	14.85^c^	0.34	3.8	<0.001
α-Linolenic acid (18:3, *n*3)	1.78^b^	2.98^a^	1.25^c^	0.05	13.1	<0.001	1.97^b^	3.43^a^	1.35^c^	0.05	10.1	<0.001
Arachidonic acid (20:4, *n*6)	3.10^a^	3.25^a^	2.37^b^	0.19	33.4	0.012	1.90	2.12	1.95	0.12	33.0	0.405
Linolenic acid (18:3, *n*6)	0.26^a^	0.24^a^	0.20^b^	0.01	13.9	<0.001	0.28^a^	0.26^a^	0.20^b^	0.01	13.7	<0.001
Eicosadienoic acid (20:2)	0.30	0.28	0.17	0.04	70.9	0.077	0.22	0.24	0.14	0.03	64.3	0.064
Dihomo-y-linolenic acid (20:3, *n*6)	0.13	0.20	0.15	0.03	–	0.288	0.10	0.15	0.14	0.03	98.9	0.378
Eicosatrienoic acid (20:3, *n*3)	0.45	0.42	0.41	0.03	24.5	0.573	0.33	0.34	0.34	0.01	20.9	0.786
Timnodonic acid (20:5, *n*3)	0.29	0.27	0.27	0.05	–	0.955	0.14	0.10	0.17	0.02	44.7	0.051
Clupanodonic acid (22:6)	0.63^a,b^	0.75^a^	0.57^b^	0.05	40.5	0.039	0.37^b^	0.52^a^	0.42^a,b^	0.04	41.7	0.036
*Saturated* (SFA)												
Myristic acid (14:0)	0.41^a^	0.33^b^	0.32^b^	0.01	12.1	<0.001	0.42^a^	0.33^b^	0.31^b^	0.01	10.6	<0.001
Pentadecanoic acid (15:0)	0.12^a^	0.12^a^	0.08^b^	0.01	55.7	0.039	0.09^a,b^	0.10^a^	0.07^b^	0.01	51.0	0.018
Palmitic acid (16:0)	19.83^a^	17.95^b^	17.07^c^	0.19	3.8	<0.001	20.3^a^	17.98^b^	17.26^c^	0.17	3.4	<0.001
Margaric acid (17:0)	0.27^b^	0.30^a^	0.20^c^	0.01	19.8	<0.001	0.27^b^	0.31^a^	0.20^c^	0.01	9.9	<0.001
Stearic acid (18:0)	6.69^a^	6.73^a^	5.58^b^	0.11	7.4	<0.001	6.35^a^	6.21^a^	5.37^b^	0.08	7.9	<0.001
Arachidic acid (C 20:0)	0.09	0.09	0.08	0.01	58.9	0.765	0.09	0.11	0.09	0.01	31.6	0.494
Lignoceric acid (24:0)	0.92^a^	0.85^a^	0.67^b^	0.05	36.6	0.01	0.56	0.56	0.49	0.03	27.6	0.347
Cerotic acid (26:0)	0.02	0.01	0.01	0.00	–	0.16	0.017^a,b^	0.02^a^	0.01^b^	0.00	–	0.010
O/L	1.23^b^	0.82^c^	3.30^a^	0.06	10.0	<0.001	1.25^b^	0.80^c^	3.43^a^	0.06	9.6	<0.001

1 NO-EE SB, normal oleic extruded expeller soybean; NO-FF SB, normal oleic full-fat soybean; HO-FF SB, high oleic full-fat soybean. Means correspond to 40 breast samples per treatment, 4 per replicate pen with broilers within one standard deviation of the average pen weight at 47 d.

a–c Means that do not share superscript letters in a row are significantly different (*P <* 0.05) by Tukey's test.

Linoleic, alpha-linolenic, and linolenic acid contents in both breast muscles were reduced (*P* < 0.001) in broilers fed HO-FF SB compared to both NO-FF and NO-EE treatments. Broilers fed HO-FF SB had breast fillets with 72.5% less linoleic acid content than fillets from NO-EE, or 2.2 times less when compared with broilers fed NO-FF SB. The HO-FF SB also reduced arachidonic acid in approximately one-third of the observed in broilers fed the other 2 treatments. The SFA like palmitic, stearic, myristic, margaric, pentadecanoic, and lignoceric acids (*P <* 0.05) were also reduced by feeding diets with HO-FF SB. Finally, no significant effect of diets (*P* > 0.05) were observed on meat content of pentadecanoic (15:1, *cis*), heptadecenoic, eicosadienoic, dihomo-y-linolenic, eicosatrienoic, timnodonic, arachidic, and cerotic acids.

## DISCUSSION

### Live performance

Broilers in the NO-EE treatment group were heavier, having faster BW gain during the first 35 d of the feeding trial than NO-FF and HO-FF broilers. At 47 d, the response was similar, but the effects in growth were nonsignificant. However, the final adjusted FCR was 7 points worse for the HO-FFSB than the NO-EE SB and the NO-FF SB was intermediate. In this experiment, broilers fed diets containing NO-EE had the greatest FI relative to the NO-FF treatment group, with similar FI values for broilers fed the HO-FF dietary treatment during the first 35 d. At 47 d, the effect of treatments was not significant on FI, probably due to high variability among pens in the last weeks, but broilers fed diets containing HO-FF SB consumed 261 g more feed than broilers fed NO-FF SB. This difference in FI affected the total consumption of AA and energy, however, HO-FF SB had the worst FCR and lower carcass yield.

These results suggested that NO-EE SB might serve as a better source of AA and energy for broilers than the FFSB. Several factors may explain these results. The higher level of fat in the FFSB products caused relatively lower pellet durability (8% points lower), which may have affected FI. In the present experiment, the diets were formulated to be isoenergetic, but our energy estimations may have either overestimated both FF-SB meal sources or underestimated the energy in NO-EE. Diets containing NO-EE may have slightly lower fiber and TIA (Table 3).

The TIA in the diets should be considered when comparing sources of SBM with different processing methods. Clarke and Wiseman (2005,^2007^) concluded that TIA should not exceed 4.0 mg/g. In our experimental diets, TIA in all diets (Table 3) was under the maximum limit, but NO-FF and HO-FF had 0.48 to 1.31 mg/kg higher TIA levels than NO-EE. Consequently, this could be one of the factors influencing the effects on broiler live performance when fed the FF SB diets compared to broilers fed NO-EE. Hoffman et al*.* (2019) observed a linear improvement in FCR when TIA decreased below 1.0 mg/g without impairing growth performance while there was excessive protein heat damage.

Subuh et al. (2002) concluded that extruded FFSB could replace SE-SBM without compromising early BW, FCR, and mortality. However, their study showed that dietary inclusion of FFSB significantly affected BW and FCR at 42 d. The FFSB used in that study was processed by passing soybeans through a roller mill and subsequently dry extruding without steam. These authors discussed that the actual ME of the FFSB may have been misestimated using the formula to calculate its energy. They discussed that the equation should account for the rolling of beans before extrusion. This grinding step could increase cellular disruption and subsequent release of oil from individual cells. The FFSB products in the present experiment were also ground prior to extrusion. More recently, Jahanian and Rasouli (2016) also demonstrated that replacing all SE-SBM with extruded FFSB significantly increased BW and FI, but FCR was worse in 5-wk-old chickens. Then, issues to obtain similar FCR are common when using FFSB, and this is an area for future investigation.

Higher dietary oleic acid elevates insulin and glucagon levels, decreases arachidonic acid and proinflamatory metabolites (Miklankova et al., 2022), and influences molecular pathways in mitochondrial and endothelial functioning (Rehman et al., 2020). Some of these effects are considered positive for animal and human health, but they may limit growth of some tissues in fast-growing animals with high use of glucose like broilers. For example, Slaughter et al. (2019) observed lower (*P* < 0.001) FI and BW in chickens fed corn-soy diets with HO oil, but no differences (*P* > 0.05) on FCR, carcass or breast yield were observed with broilers fed similar diet with NO oil.

### Carcass and component part yields

In the present experiment, only carcass yield was reduced (0.88% points) in HO-FF SB compared to NO-EE SB, while the yields of each carcass part were unaffected by dietary treatments. In addition, dietary treatments did not significantly affect the relative weights of abdominal fat. The diets were formulated to be isocaloric and isonitrogenous, but the digestibility values for oleic acid have been reported to be lower than linoleic acid in experiments with HO sunflower (Rodríguez et al., 2005). These HO meals also have lower fat digestibility and AMEn. Consequently, the HO-FF could yield less energy limiting nutrient utilization and carcass development. However, the diet balance did not cause adverse effects on carcass traits that could be detected.

The FFSB in properly balanced diets can effectively replace other sources of SBM without affecting carcass or part yield. In a study conducted by Subuh et al. (2002), SE-SBM was replaced with extruded FFSB at levels ranging from 0 to 100% with intervals of 25% in broiler diets. No significant effect of this dietary replacement was observed on the dressing percentage or abdominal fat percentage on broilers raised to 42 d. Even increasing the dietary energy level did not adversely affect these traits. However, in their study, CP and AA levels remained constant with dietary energy levels. Powell et al. (2011) did not detect a significant difference in the carcass weight of broiler chickens fed diets containing either SE-SBM or EE SB. Alsaftli et al. (2015) also observed no significant effect of extruded FFSB on carcass yield, breast and thigh parts including bone, and relative liver weight to live weight of female turkeys when SE-SBM was replaced with 10, 15, and 20% extruded FFSB.

Recently, Janocha et al. (2022) showed that replacing the SE-SBM with EE SB and extruded FFSB in broiler diets increased (*P <* 0.05) preslaughter BW at 42 d. Although there was no effect of dietary treatment on dressing percentage, broilers fed the SE-SBM and EE SB cake showed a higher breast and leg yield (by 4.74 and 7.54%) and lower abdominal fat (by 31.1%) and skin with subcutaneous fat (by 18.8 and 13.4%) in comparison to chickens from the extruded FFSB group (*P <* 0.05).

The present study showed no effects of dietary treatments on the liver, pancreas, intestine, gizzard, proventriculus, and spleen weights. Pacheco et al. (2014) did not observe an effect of extruded SB seeds on gizzard weight percentage in chicken carcasses. Śliwa and Brzóska (2018) did not observe any impact on giblets (heart, liver, and gizzard) when, 10, 18, and 40% of SB expeller cake was used in broiler chicken feed instead of SE-SBM.

### Poultry meat quality parameters

Meat quality is a term that aims to represent the various intrinsic and extrinsic variables driving a customer's valuation of a meat product. The quantifiable properties of meat such as water holding capacity, dripping and cooking loss, pH, and color were evaluated. No significant effect on pH was observed in breast meat at 6 h postslaughter, but at 24 h a significant treatment effect was observed. The results from the present experiment fall within the optimal pH range of 5.35 to 6.10 for poultry meat 24 h postslaughter also known as ultimate pH, as indicated by Rycielska et al. (2010). Alterations in poultry meat pH are due to postmortem muscle glycolysis and increased content of lactic acid. However, myopathies and variations on muscle energy metabolism may also affect ultimate pH (Baldi e*t* al., 2020).

This effect of soybean sources on ultimate pH has been observed by others (Milczarek and Osek, 2019; Janocha et al., 2022). Janocha et al. (2022) found no significant differences in broiler breast muscles’ pH 15 mins after slaughtering among birds fed SE-SBM, EE SB, and FFSB. However, there were measurable differences between thigh muscles 24 h after slaughter. The pH was 5.81 in broilers fed extruded FFSB compared to 5.88 and 5.95 for EE SB cake and SE SBM, respectively. The metabolic and physiological mechanisms that explain this impact of SBM sources on meat pH should be better understood with further research.

The 2 most severe anomalies in chicken breast muscle reported recently are WB and WS (Kuttappan et al., 2016; Meloche et al., 2018; Petracci et al., 2019), which lead to unattractive appearance for consumers, carcass downgrading, condemnation, and reduced protein functionality in processed poultry meat products (Mudalal et al., 2014; Petracci et al., 2014; Tijare et al., 2016; Petracci et al*.,* 2019). Despite the increased incidence of WB and WS in broilers, the exact reason for these muscle myopathies remains unknown (Khan et al., 2021). The relatively new muscle myopathy SM similarly occurs for unknown reasons. No significant effect of dietary SBM type was observed (*P >* 0.05) on myopathies, including WB, WS, and SM incidence. However, broilers fed diets containing NO-FF had more (17.50%) normal breast (WB score 1), and a tendency (*P* = 0.075) to lower WB incidence rate (2.48) than broilers fed diets with other SB sources (Table 9). The final BW and BW gain differences in the last period were not significant (*P* > 0.05) to consider growth rate the only factor responsible for this effect in myopathies. Other research groups have observed effects on meat quality depending on the SBM source (Milczarek and Osek, 2019; Janocha et al., 2022), and the explanation is still not clear.

### Fatty acid profile

The primary fatty acids in chicken meat include oleic acid, palmitic acid, linoleic acid, stearic acid, and arachidonic acid (Zhao et al., 2011; Amorim et al., 2016). A dietary requirement for essential fatty acids for poultry is usually expressed only for linoleic acid, and 10 g/kg is believed to be satisfactory for optimal performance (NRC, 1994). This quantity is expected to be contributed by the native triglycerides in conventional poultry diets (Zollitsch et al., 1997). In the current experiment, broilers fed the HO-FF had higher oleic acid content in their *Pectoralis* muscles compared to the other 2 treatments.

Slaughter et al. (2019) fed broilers a corn-soy diet with either SE-SBM with NO oil or HO SBM with HO oil. At 42 d, the breast and thigh meat were analyzed for fatty acid composition. The analysis demonstrated that dietary supplementation of HO oil increased (*P* < 0.001) the content of MUFA but decreased the contents of PUFA and SFA in breast and thigh meat relative to the corn-soy diet with NO oil.

In the current experiment, the HO-FF SBM increased the proportion of oleic acid by 55 to 86% in *Pectoralis major* and *minor* muscles, while linoleic acid was reduced by 42 and 54% in comparison to the NO-EE and NO-FF treatment groups, respectively. Dietary MUFA have been associated with reduced risk factors for metabolic syndrome and cardiovascular diseases and may foster a healthy blood lipid profile, lower blood pressure, enhance insulin sensitivity, control blood glucose levels, and decrease the risk of obesity (Gillingham et al., 2011).

Studies have shown that dietary fatty acid composition positively correlates to meat fatty acid composition in monogastric animals (Semwogerere et al., 2019). Breast meat samples from broilers fed diets containing HO peanuts had higher levels of MUFA (55%) than those (35%) fed on a traditional corn-soy diet (Toomer et al., 2020a). Additionally, broilers fed on an HO peanut diet had significantly reduced SFA (palmitic and stearic) and *trans* fatty acid content in their breast fillets compared to the NO controls (Toomer et al., 2020a). Studies have shown that palmitic and stearic acid are the most consumed dietary SFA in Western diets associated with increased cholesterol and risk for cardiovascular disease (Rooijen and Mensink, 2020). Then, reducing these fatty acids should be positive for healthier poultry products.

## CONCLUSIONS

The soybean processing methods modify its nutrient composition and can affect broiler chickens’ live performance and carcass yield. Broilers fed diets containing HO-FF SB had similar BW, worse FCR (7 points), and 0.88% points lower carcass yield than broilers fed diets containing NO-EE at 47 d of age. No significant differences were observed in 47 d BW, adjusted FCR, and carcass yield between broilers fed diets including NO-FF SB and HO-FF SB. The SBM sources did not affect total (0–47 d) feed intake, the carcass cut-up parts yield, abdominal fat, and visceral organs. There were also no differences between diets for meat quality parameters such as initial pH, color, dripping, and cooking loss. However, broilers fed the HO-FF diet had 55% higher levels of oleic acid in their breast meat than broilers fed NO-EE and NO-FF diets, as well as lower PUFA (linolenic acid and alpha-linolenic acid) and SFA (palmitic and stearic acid) contents relative to the other treatments.

The release of numerous HO oilseed cultivars may influence the use of these meals within animal feed production as a dietary means to enrich poultry meat with MUFA while reducing SFA content. However, additional poultry-feeding trials must be conducted to improve live performance in poultry-fed HO oilseed diets. This study demonstrated the efficacy of HO beans to improve the fatty acid profile of broiler meat.
